# Ecology of a polymetallic nodule occurrence gradient: Implications for deep‐sea mining

**DOI:** 10.1002/lno.11157

**Published:** 2019-03-13

**Authors:** Erik Simon‐Lledó, Brian J. Bett, Veerle A. I. Huvenne, Timm Schoening, Noelie M. A. Benoist, Daniel O. B. Jones

**Affiliations:** ^1^ National Oceanography Centre University of Southampton Southampton UK; ^2^ Ocean and Earth Science, National Oceanography Centre University of Southampton Southampton UK; ^3^ Marine Geosystems Working Group, GEOMAR Helmholtz Centre for Ocean Research Kiel Germany

## Abstract

Abyssal polymetallic nodule fields constitute an unusual deep‐sea habitat. The mix of soft sediment and the hard substratum provided by nodules increases the complexity of these environments. Hard substrata typically support a very distinct fauna to that of seabed sediments, and its presence can play a major role in the structuring of benthic assemblages. We assessed the influence of seafloor nodule cover on the megabenthos of a marine conservation area (area of particular environmental interest 6) in the Clarion Clipperton Zone (3950–4250 m water depth) using extensive photographic surveys from an autonomous underwater vehicle. Variations in nodule cover (1–20%) appeared to exert statistically significant differences in faunal standing stocks, some biological diversity attributes, faunal composition, functional group composition, and the distribution of individual species. The standing stock of both the metazoan fauna and the giant protists (xenophyophores) doubled with a very modest initial increase in nodule cover (from 1% to 3%). Perhaps contrary to expectation, we detected little if any substantive variation in biological diversity along the nodule cover gradient. Faunal composition varied continuously along the nodule cover gradient. We discuss these results in the context of potential seabed‐mining operations and the associated sustainable management and conservation plans. We note in particular that successful conservation actions will likely require the preservation of areas comprising the full range of nodule cover and not just the low cover areas that are least attractive to mining.

Abyssal polymetallic nodule fields represent a unique deep‐sea habitat (Radziejewska 2014). The hard substratum provided by the nodules combined with the background soft sediment seabed acts to increase habitat complexity and is thought to promote the occurrence of some of the most biologically diverse seafloor assemblages in the abyss (Amon et al. 2016; Gooday et al. 2017). This unusual and diverse habitat is potentially subject to imminent large‐scale human impacts in the form of seafloor mining (Wedding et al. 2015; Kuhn et al. 2017). Mining disturbances are likely to extend over extremely large seafloor areas (Aleynik et al. 2017) and have a clear potential to drive major changes in the resident fauna (Jones et al. 2017). Predicting the nature of such changes remains difficult; the ecology of this remote habitat is poorly understood, in particular, very little is known of the biodiversity associated with nodules (Veillette et al. 2007; Vanreusel et al. 2016).

The presence of hard substratum is thought to be a key factor in structuring heterogeneous deep‐sea habitats (Buhl‐Mortensen et al. 2010; Bell et al. 2016). For example, modest variations in the availability and the composition of hard surfaces can influence the larval settlement processes of the seafloor fauna (Van Dover et al. 1988; Roberts et al. 2006). Substratum selectivity is commonly exhibited by many deep‐sea species, including soft corals (Sun et al. 2011), sponges (Lim et al. 2017), and foraminifera (Gooday et al. 2015). The presence and extent of hard substratum is therefore expected to exert a significant control on the composition of deep‐sea benthic assemblages (Levin et al. 2001; Smith and Demopoulos 2003). Seafloor environments in the deep sea with extensive hard substratum range in nature from landscape‐scale features such as seamounts (Clark et al. 2010) and canyons (De Leo et al. 2010) to widely dispersed pebbles, cobbles, and boulders referred to as iceberg drop‐stones (Meyer et al. 2016) and the similar human artifact habitat produced by steamship clinker (Ramirez‐Llodra et al. 2011). While individual polymetallic nodules are generally small, 1–20 cm in diameter, nodule fields can extend over extremely large areas, many hundreds of square kilometers, as occurs in the Clarion Clipperton Zone (CCZ) of the central Pacific Ocean (Kuhn et al. 2017).

Polymetallic nodules in the CCZ are thought to support a specialized fauna that differs from that of nodule‐free sediment areas (Mullineaux et al. 1987; Gooday et al. 2015). Nodule‐dwelling meiofauna such as nematodes, tardigrades, harpacticoids, and foraminifera inhabit the crevices (Veillette et al. 2007; Miljutina et al. 2010), whereas sessile macrofauna and megafauna such as polychaetes, sponges, cnidarians, and xenophyophores are commonly found attached to nodule surfaces (Gooday et al. 2015; Amon et al. 2016). Consequently, nodule occurrence has been linked with variations in faunal standing stocks and distributions (Amon et al. 2016; Vanreusel et al. 2016). However, logistic constrains have limited the detailed monitoring of nodule cover (Amon et al. 2016; Vanreusel et al. 2016), restricting the assessment of seafloor ecology along continuous nodule occurrence gradients.

Recent advances in large‐scale seafloor visual imaging (Morris et al. 2014) coupled with automated nodule‐detection algorithms (Schoening et al. 2016; Schoening et al. 2017) now make such studies possible. Here, we combine extensive nodule coverage and faunal data obtained by photography from an autonomous underwater vehicle (AUV) to examine the effect of nodule occurrence on the megabenthos in the CCZ. We include protozoan, invertebrate, and fish species that can be distinguished in photographs, having body length scales >1 cm, as members of the megafauna. In particular, we consider variations in their standing stock, biological diversity, and faunal composition along a nodule cover gradient. This work was carried out within an “Area of Particular Environmental Interest” (APEI), a form of marine‐protected area designed as a conservation measure in response to potential future seabed mining in the region (International Seabed Authority 2012). Consequently, we also cast our results in the context of the sustainable management and conservation of this unusual abyssal habitat.

## *Methods*

### Study area

**Figure 1:**
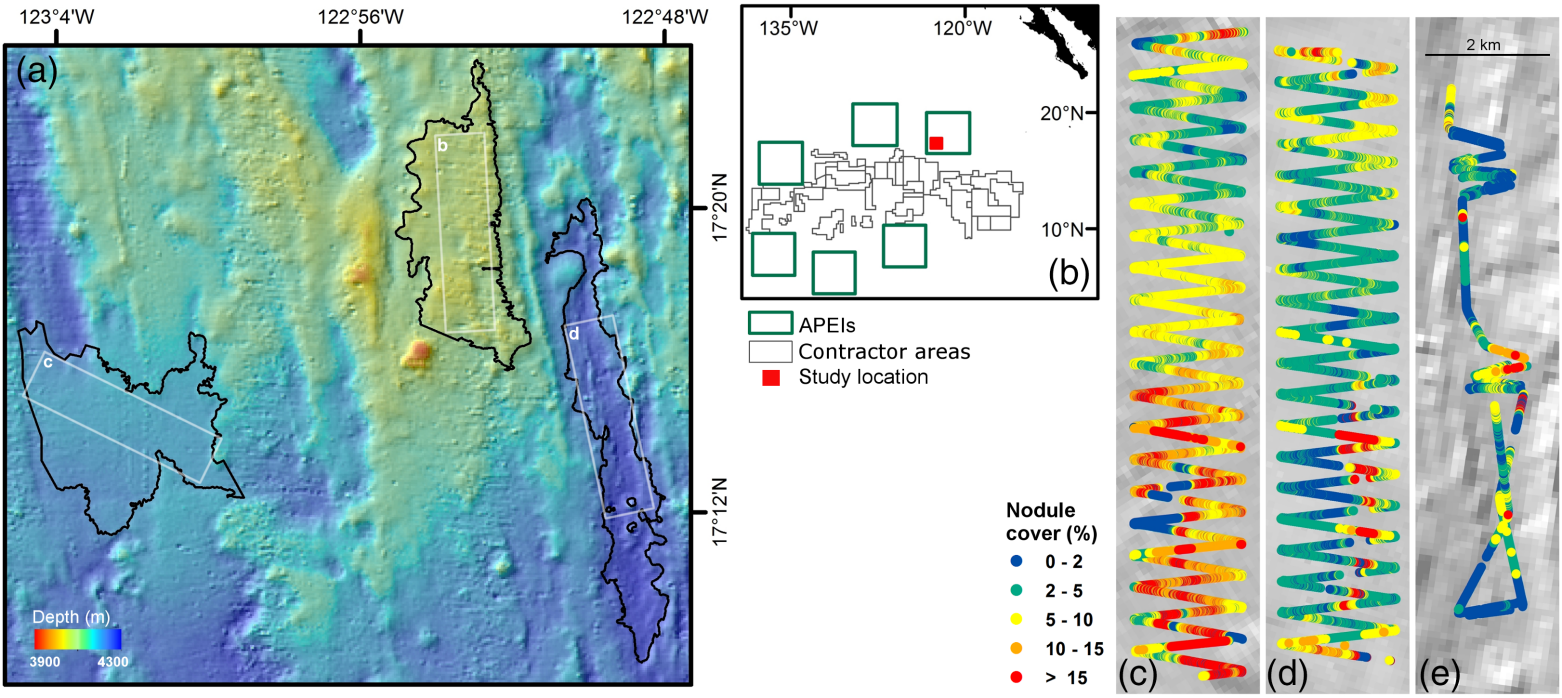
Study area location within APEI 6 in the CCZ (NE Pacific). (**a**) Bathymetric chart, with landscape types regions in heavy outline (left to right: “flat,” “ridge,” and “trough”), white rectangles indicate AUV survey areas. (**b**) Chart of the eastern CCZ with contractor areas, APEIs, and present‐study area. (**c–e**) Individual landscape type survey areas, showing AUV track color coded to seafloor nodule cover level (c, “flat”; d, “ridge”; e, “trough”).

Our study area was a 5500 km^2^ rectangular region of seafloor centered on 17°16′N 122°55′W within the APEI6 region (Fig. 1). This location was selected to have similar topographic relief to mining contract areas in the central CCZ. Water depth ranged 3950–4250 m, and the seafloor landscape comprised a succession of crenulated ridges and shallow troughs oriented north–south between dispersed level‐bottom (<3° slope) areas. General seafloor conditions are described by Simon‐Lledó et al. (2019) and are only briefly summarized here. Surface sediments (0–1 cm) were homogenous across the study area, dominated by very fine silt and clay particles (58–68% < 7.8 *μ*m diameter), and had a very low content of total organic carbon (0.44 ± SD 0.05%). The polymetallic nodules present were of a flattened, ellipsoidal form with smooth surfaces. The seafloor exposed mean individual nodule area was 2.5 cm^2^, with most nodules <5 cm^2^ (90%) and very few >10 cm^2^ (1%). In individual seafloor photographs, average nodule cover was 6.4% and ranged from nodule free to 37%. Nodule cover was patchy, with extremes of variation occurring at meter scales (Fig. 1). All results reported here were acquired in April–May 2015, during RRS *James Cook* cruise 120; additional supporting technical detail is provided by Jones (2015).

### Data collection and processing

Seafloor images were collected using a digital camera (FLIR Integrated Imaging Solutions *Grasshopper2*; 2448 × 2048 pixels) mounted vertically in the AUV Autosub6000 (Morris et al. 2014). The AUV was programmed for a target altitude of 3 m above the seafloor, a speed of 1.2 m s^−1^, and a photographic interval of 850 ms. At the target altitude, individual vertical photographs imaged 1.71 m^2^ of seabed. Three landscape types (ridge, flat, and trough), delimited by objective analysis of bathymetric data, were surveyed using zig–zag surveys with random start points (Strindberg and Buckland 2004), as detailed by Simon‐Lledó et al. (2019). A total of 40 individual image transects were surveyed in each landscape type. Images taken as the vehicle changed course, i.e., junctions between transects, were removed. In the remaining straight‐line sections, every second image was removed to avoid overlap between consecutive images and prevent double counting. To ensure consistency in specimen and nodule detection, images outside the altitude range 2–4 m were also removed. Four transects were randomly selected from each landscape type for subsequent analysis. The full resultant dataset was composed of data from 10,052 nonoverlapping images, representing a seafloor area of 18,580 m^2^.

All images were color corrected, as described by Morris et al. (2014), before manual and automated analyses were performed to obtain biological and environmental data. Nodule cover (%) was quantified using the Compact‐Morphology‐based polymetallic Nodule Delineation method (CoMoNoD; Schoening et al. 2017). The CoMoNoD algorithm calculates the size of each nodule (i.e., seafloor exposed area) detected in an image, enabling the calculation of descriptive nodule statistics. Note that it is currently not possible to directly relate the image‐based assessments of seabed nodule cover with those made by direct sampling methods (Schoening et al. 2017; Gazis et al. 2018). Megafauna specimens were identified to the lowest taxonomic level possible, and their physical dimension was measured, using BIIGLE 2.0 (Langenkämper et al. 2017). Each specimen was assigned to a “firmly nodule‐attached” (NA) or “freely mobile” (FM) life‐habit category. The biovolume of individual metazoan specimen was estimated as a proxy for biomass, using the generalized volumetric method described by N. M. Benoist et al. (unpubl.).

To ensure consistency in specimen identification, a CCZ‐standardized megafauna morphospecies catalog was updated from the taxonomic compilation developed by the International Seabed Authority (available online: http://ccfzatlas.com), which we further expanded in consultation with international taxonomic experts and by reference to existing literature (Dahlgren et al. 2016; Glover et al. 2016; Amon et al. 2017; Kersken et al. 2018; Molodtsova et al. 2017). The likely feeding behavior of each morphospecies was inferred from similar organisms described in the literature (e.g., Iken et al. 2001). The full dataset comprised 7837 metazoan specimens in 133 morphospecies and 47,133 giant foraminifera (xenophyophores) specimens in 22 morphospecies.

### Data analysis

To perform an initial broad assessment of the potential influence of seafloor nodule cover on the ecological characteristics of the megafauna, all images from the three landscape types were pooled. This total image set was then ordered by estimated nodule cover and divided into 10 subsets at nodule‐cover breakpoints chosen to yield approximately equal numbers of megafaunal observations in each subset. Metazoan and xenophyophore data were processed separately on the basis that it was not possible to determine whether the latter were living from the images (Hughes and Gooday 2004). Across the 10 resultant nodule‐cover classes, metazoan megafauna counts ranged 784–787 and xenophyophore counts 4714–4719. To establish measures of variability in ecological characteristics within the nodule‐cover classes, the corresponding image subsets were resampled using a modified form of bootstrapping (Davison and Hinkley 1997; Manly 1997). Each image subset was randomly resampled with replacement until a minimum of 500 specimens were encountered, and that process repeated 1000 times for each nodule‐cover class. This resampling process yielded bootstrap‐like samples that ranged in metazoan specimen counts 500–565 (490–1082 images) and xenophyophore counts 500–587 (70–230 images). We adopted these specimen‐count–based methods to recognize and control the impact of specimen number on the estimation of biological diversity and faunal composition parameters (Sanders 1968; Forcino et al. 2015; Simon‐Lledó et al. 2019).

A range of ecological parameters was calculated for each of the 10 × 1000 bootstrap‐like samples, including metazoan and xenophyophore numerical density (ind m^−2^) and metazoan biovolume density (mL m^−2^ ≈ g fresh wet weight [fwwt] m^−2^). To examine the range of diversity characteristics, Hill's diversity numbers of order 0, 1, and 2 (Hill 1973; Jost 2006) were calculated as metazoan morphospecies richness (*S*_N_), the exponential form of the Shannon index (exp *H'*), and the inverse form of Simpson's index (1/*D*), each of which we report as numbers of morphospecies. We also calculated morphospecies density (*S*_A_), based on an additional set of bootstrap‐like samples generated following the same procedure, but with a controlled seabed area encompassed by each sample (650–670 m^2^; 149–395 metazoan specimens). Variation in metazoan community composition was assessed by two‐dimensional nonmetric multidimensional scaling (nMDS) ordination of all 10 × 1000 bootstrap‐like samples, based on square‐root–transformed faunal density and use of the Bray–Curtis dissimilarity measure (Clarke 1993).

Mean (median in the case of biovolume assessment) values of these various parameters were calculated from each bootstrap‐like sample set, together with corresponding 95% confidence intervals based on the simple percentile method, a nonparametric approach (Davison and Hinkley 1997). Data processing and analyses were performed using a custom R (R Core Team, 2014) script incorporating multiple functions from the “vegan” package (Oksanen et al. 2018). In addition to the general analyses of ecological responses to the nodule cover gradient, we considered landscape‐type–related variations in those responses by undertaking separate analyses within each landscape type (Supporting Information section S1).

We report statistical assessments of variations in ecological parameters along the nodule cover gradient in two forms: (1) by nonparametric correlation with nodule cover (Spearman's rank correlation) and (2) by comparisons of the 95% confidence interval of the minimal nodule cover class with those of all other nodule cover classes. Note that the latter is undertaken as a single assessment, not a pairwise examination, i.e., the upper limit of the minimal nodule class must be lower than the lower limits of all other nodule cover classes. In such cases, we report this as significant at *p* < 0.05, although the true (undetermined) *p* value will, necessarily, be considerably lower. Similarly, we do not formally test for the occurrence of unimodal responses and simply describe (and illustrate) them when they are visually apparent.

## *Results*

### Standing stocks

**Figure 2:**
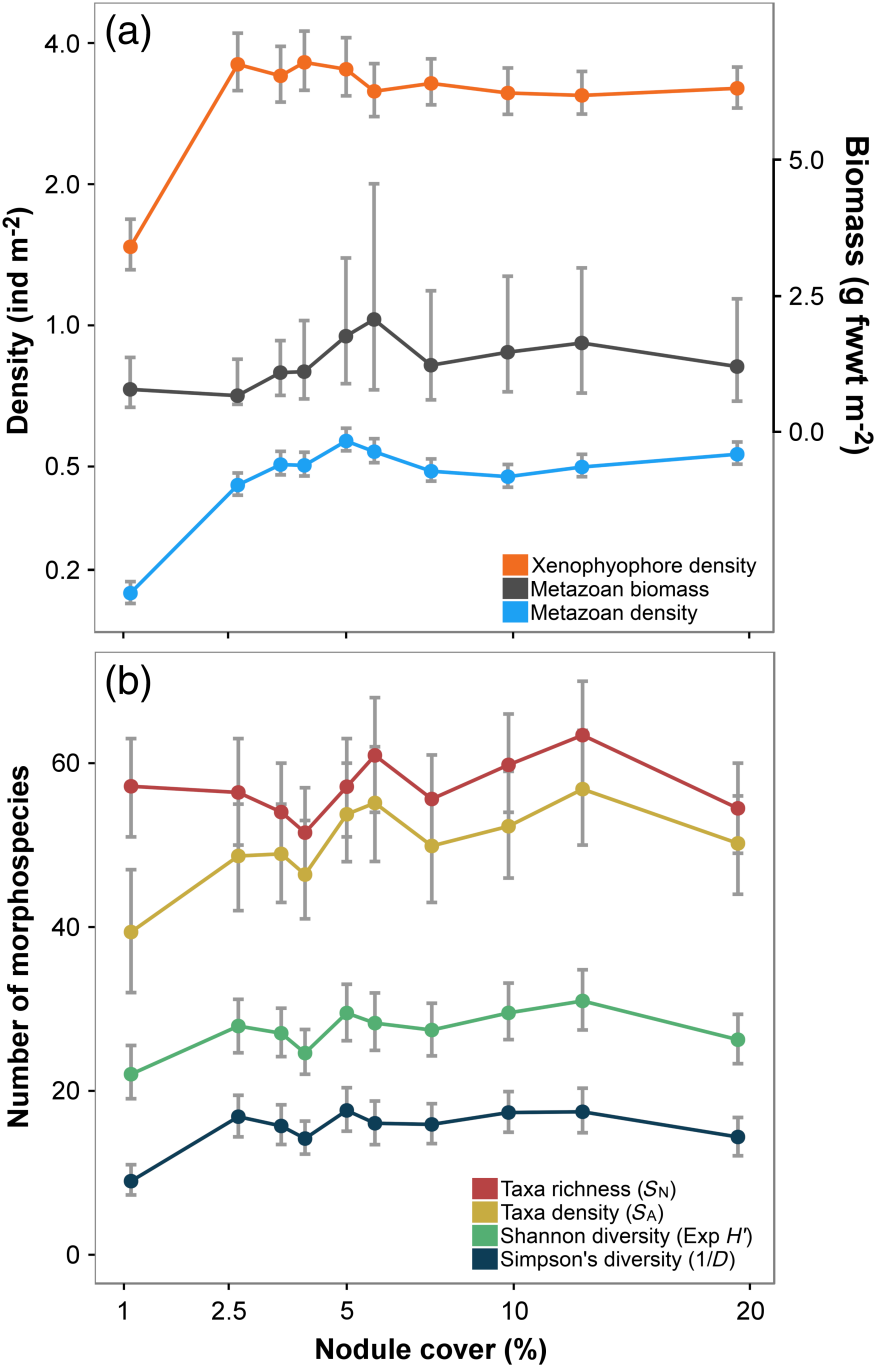
Variation in (**a**) standing stock and (**b**) diversity with seafloor nodule cover. Points indicate mean (median for metazoan biomass) values of each parameter calculated from each nodule‐cover class, error bars represent 95% confidence intervals. (**a**) Numerical density of metazoans and xenophyophores (left axis) and metazoan biomass density (right axis). (**b**) Metazoan diversity measures: morphospecies richness (*S*_N_), morphospecies density (*S*_A_), exponential Shannon index (exp *H*′), and inverse Simpson's index (1/*D*).

Metazoan and xenophyophore density were substantially, and statistically significantly (*p* < 0.05), lower in the lowest nodule‐cover class (Fig. 2a). We found no statistically significant correlation (Spearman's rank) between density and nodule availability (Table 1); density variation of both groups across the nodule gradient described a rapid asymptote, stabilizing in cover levels >2–3%. In contrast, metazoan biomass density showed no statistically significant variations or correlation with nodule cover gradient (Fig. 2a).

**Table 1:** Variations in ecological parameters with seafloor nodule cover. Summary results of tests performed between mean (median for metazoan biomass density) values of each parameter calculated from nodule‐cover classes, indicating occurrence of statistically significant difference between nodule class 1 (mean cover, 1.1%) and all other classes (cover > 2%), and Spearman's rank correlation (r_s_). In bold: significance level *p* < 0.05

	Distinct class 1	Correlation	
		*r*_s_	*p* value
*Standing stock* (ind m^−2^)			
Xenophyophores	Yes	−0.297	0.404
Metazoans	Yes	0.345	0.328
Metazoan biomass (g fwwt m^−2^)	No	0.624	0.053
NA metazoans	Yes	0.466	0.174
FM metazoans	Yes	0.224	0.533
Porifera msp‐5	No	−0.976	**<0.001**
*Callozostron bayeri* sp. inc.	No	−0.600	0.067
*Columnella* sp. indet.	Yes	0.760	**0.011**
*Lepidisis* sp. indet.	No	0.952	**<0.001**
*Diversity and composition*			
Morphospecies richness (*S*_N_)	No	0.248	0.405
Morphospecies density (*S*_A_)	No	0.721	**0.018**
Exponential Shannon (exp *H*′)	No	0.478	0.161
Inverse Simpson (1/*D*)	Yes	0.345	0.328
nMDS‐dimension 1	Yes	0.891	**0.001**

### Biological diversity

Diversity measures calculated from samples with controlled number of individuals exhibited no statistically significant correlation with nodule cover (Table 1). Morphospecies richness (*S*_N_) was near‐constant across nodule‐cover classes with no indication of any significant difference between any pair of classes (Fig. 2b). The Shannon index (exp *H′*) was more variable across nodule‐cover classes but exhibited no coherent substantive change along the nodule gradient. In contrast, Simpson's index (1/*D*) showed a statistically significant (*p* < 0.05) lower value in the minimum nodule cover class compared to all other classes. Morphospecies density (*S*_A_), as calculated from controlled area samples, was statistically significantly correlated with nodule cover (Table 1).

### Faunal composition

#### *Taxonomic groups*

**Figure 3:**
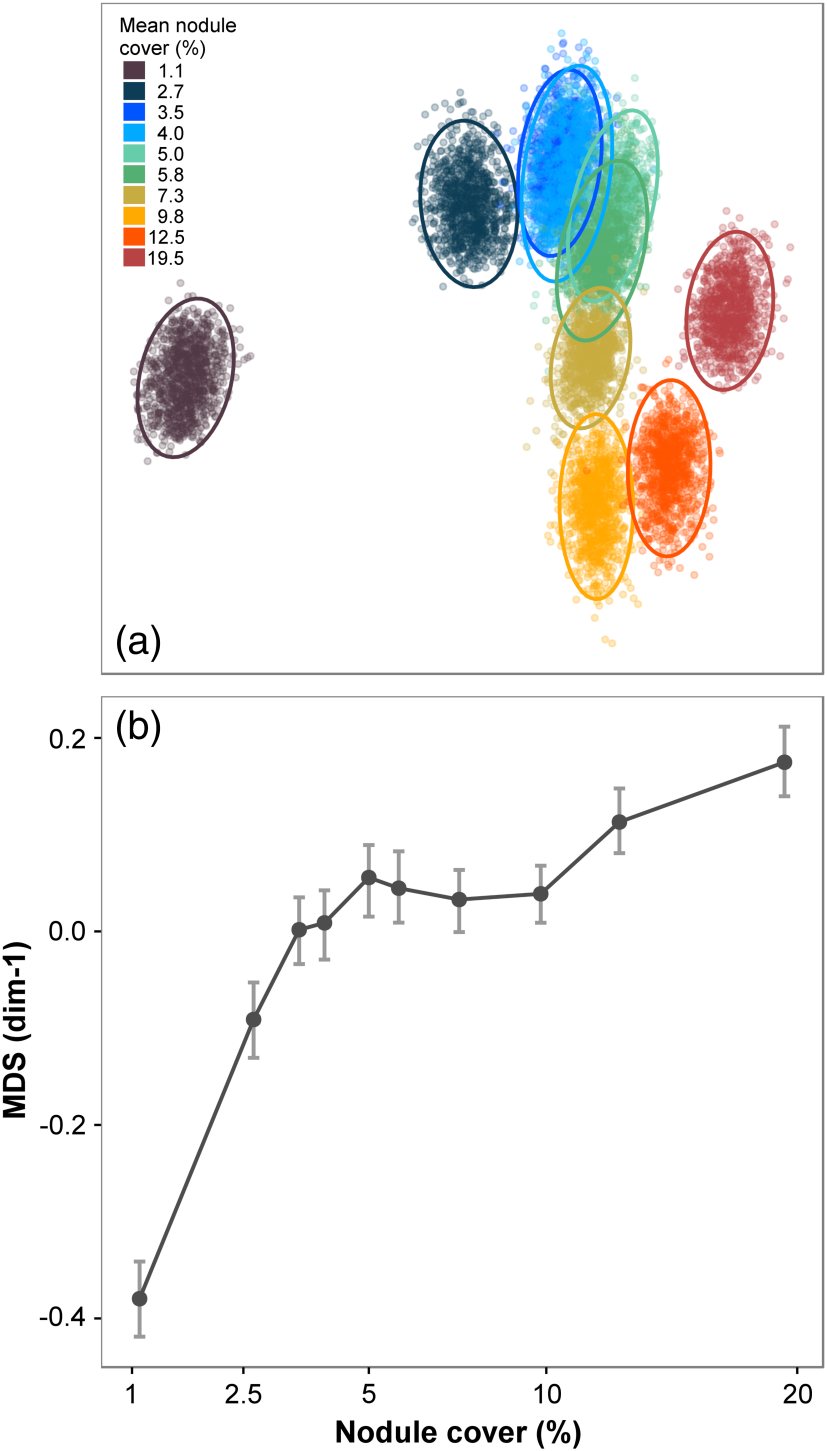
Variation in community composition with seafloor nodule cover. (**a**) Two‐dimensional nMDS ordination plot illustrating results from all bootstrap‐like samples, ellipses represent 95% confidence intervals. (**b**) Variation in nMDS‐dimension 1 with seafloor nodule cover, illustrated as mean and 95% confidence intervals.

**Figure 4:**
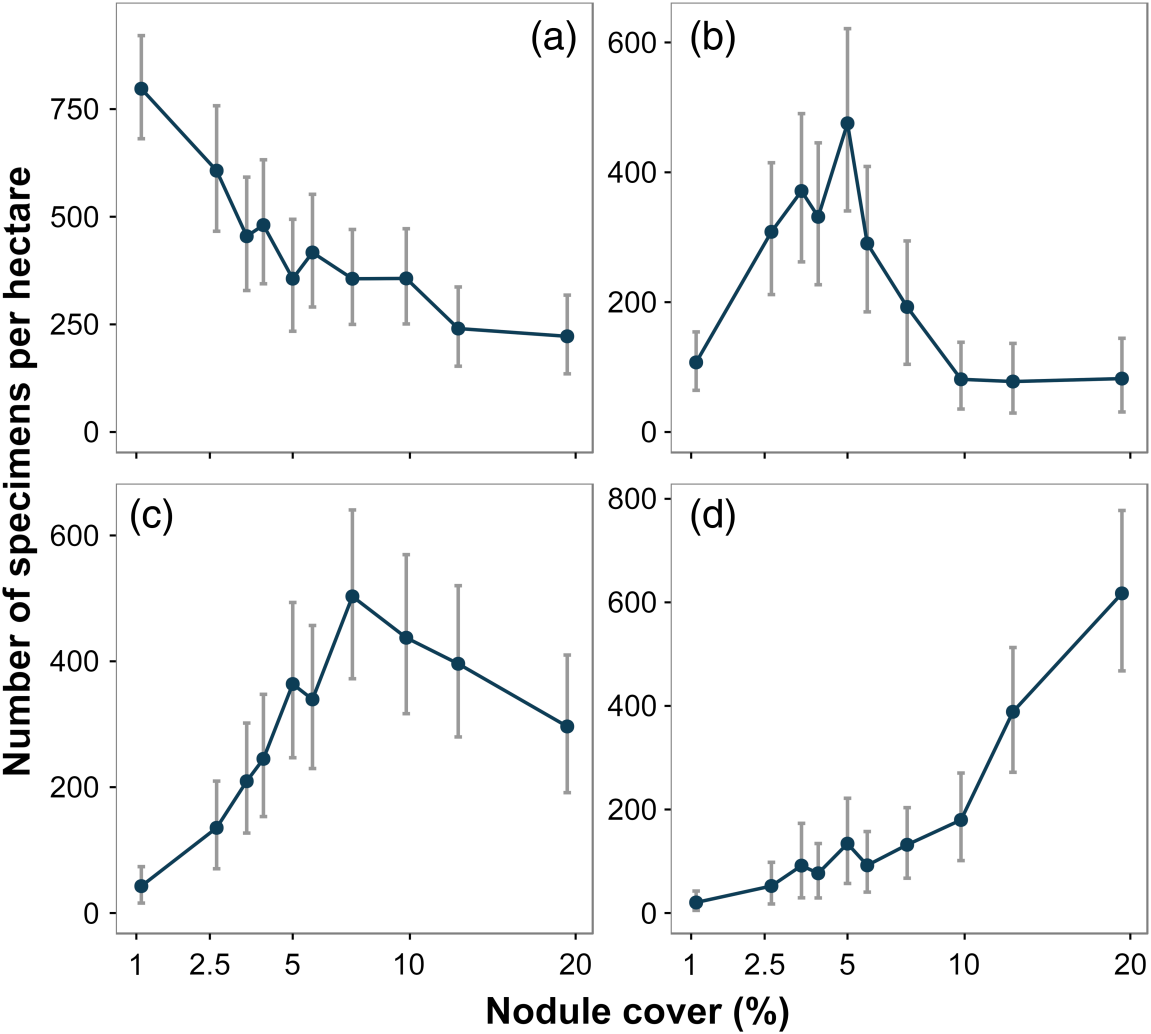
Variation in selected metazoan morphospecies density with seafloor nodule cover. (**a**) Sponge: Porifera msp‐5. (**b**) Primnoid soft‐coral: *Callozostron bayeri* sp. inc. (**c**) Bryozoan: *Columnella* sp. indet. (**d**) Bamboo soft‐coral: *Lepidisis* sp. indet., illustrated as mean and 95% confidence intervals.

Two‐dimensional MDS ordination of bootstrap‐like controlled count samples showed that metazoan assemblage composition progressively changed along the nodule gradient (Fig. 3a). MDS‐dimension 1 values were strongly and statistically significantly correlated with nodule cover (*r*_s_ = 0.891, *p* = 0.001; Table 1). In addition, MDS‐d1 values in the minimum nodule‐occurrence class were substantially and statistically significantly different from all other nodule‐cover classes (Fig. 3b). Among the 15 most abundant morphospecies (Supporting Information Fig. S2‐3), a graded series of distributions across nodule‐cover classes was apparent (Supporting Information Fig. S2‐4; Table S2‐1). For example (Fig. 4), (1) monotonic decline with nodule cover, Porifera msp‐5, strong and statistically significant correlation with nodule cover (*r*_s_ = −0.976, *p* < 0.001; Table 1); (2) unimodal with nodule cover, *Callozostron bayeri* sp. inc. and *Columnella* sp. indet.; and (3) monotonic increase with nodule cover, *Lepidisis* sp. indet., strong and statistically significant correlation with nodule cover (*r*_s_ = 0.952, *p* < 0.001; Table 1). The densities of Polychaete msp‐5 and Actiniaria msp‐18 were statistically significantly and substantially lower in the lowest nodule‐cover class, whereas those of *Ophiosphalma* sp. indet. and Irregularia msp‐1 were also lower in the lowest nodule‐cover class, although marginally (Supporting Information Fig. S2‐4). Among major taxa, a graded series of distributions across nodule cover classes was also apparent (Supporting Information Fig. S2‐2; Table S2‐1).

#### *Functional groups*

**Figure 5:**
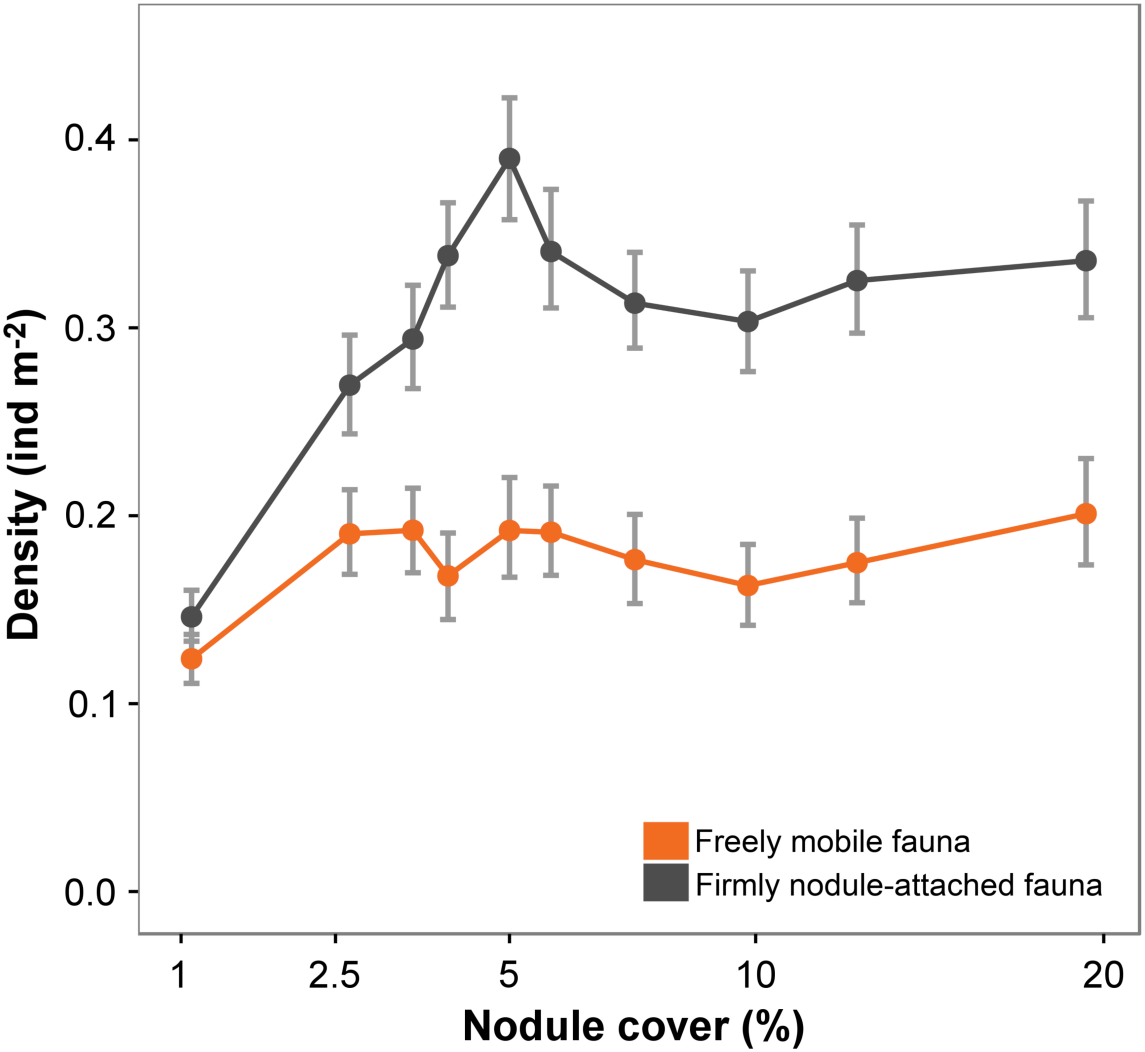
Variation in metazoan density for different life modes with seafloor nodule cover, illustrated as mean and 95% confidence intervals.

Neither NA metazoans nor FM metazoans exhibited variations in faunal density that were statistically significantly correlated with nodule cover (Table 1). However, in both cases, density in the lowest nodule‐cover class was significantly lower than in any other class (Fig. 5). Both deposit‐feeder and suspension‐feeder faunal density was statistically significantly and substantially lower in the lowest nodule‐cover class, whereas predator and scavenger density showed no statistically significant variations across the nodule cover gradient (Supporting Information Fig. S2‐1). Variation in suspension and deposit‐feeder density across the nodule gradient described a rapid asymptote, yet none of the three feeding group's densities exhibited a statistically significant correlation with nodule cover (Supporting Information S2, Table S2‐1).

## *Discussion*

We found substantial and statistically significant variations in megafaunal standing stock and faunal composition along a gradient of seafloor nodule cover; corresponding variation in diversity measures was modest. These responses were generally graded with nodule cover. However, in many cases, the magnitude of change between the first two cover classes was particularly marked. Both of these observations are of direct relevance to sustainable management and conservation concerns in relation to seabed mining in the CCZ and similar environments elsewhere.

### Standings stocks

Differences in metazoan density across the nodule cover gradient were predominately driven by variations in suspension feeder abundance, particularly anthozoans (i.e., Actiniaria and Alcyonacea) living attached to nodules; the densities of which were substantially and statistically significantly reduced in the lowest nodule class (Supporting Information Figs. S2‐1, S2‐2b). Hard substrata provide a stable anchor point for suspension feeders and enable the placement of food‐catching structures into faster off‐bottom currents (Wildish and Kristmanson 2005). Enhanced densities of hard substratum attached fauna have been observed on bedrock in seamounts or canyons (Clark et al. 2010; Baker et al. 2012; Jones et al. 2013), in areas with drop‐stones (Jones et al. 2007; Meyer et al. 2016), and in polymetallic nodule fields (Amon et al. 2016; Vanreusel et al. 2016). Our results provide additional detail that suggests a nonlinear, asymptotic relationship between standing stock and nodule cover (Fig. 2a). This response may be explained by resource limitation (Tilman 1982), i.e., hard substratum is initially limiting, but food resource (i.e., advecting organic particles) becomes limiting as attached suspension feeder density increases (Jeffreys et al. 2009). Variation in suspension‐feeder density at the landscape‐type scale sustains this hypothesis and suggest that the transition between limiting resources (i.e., from nodules to food), in the present case, occurs at nodule cover > 5% (Fig. 5).

**Figure 6:**
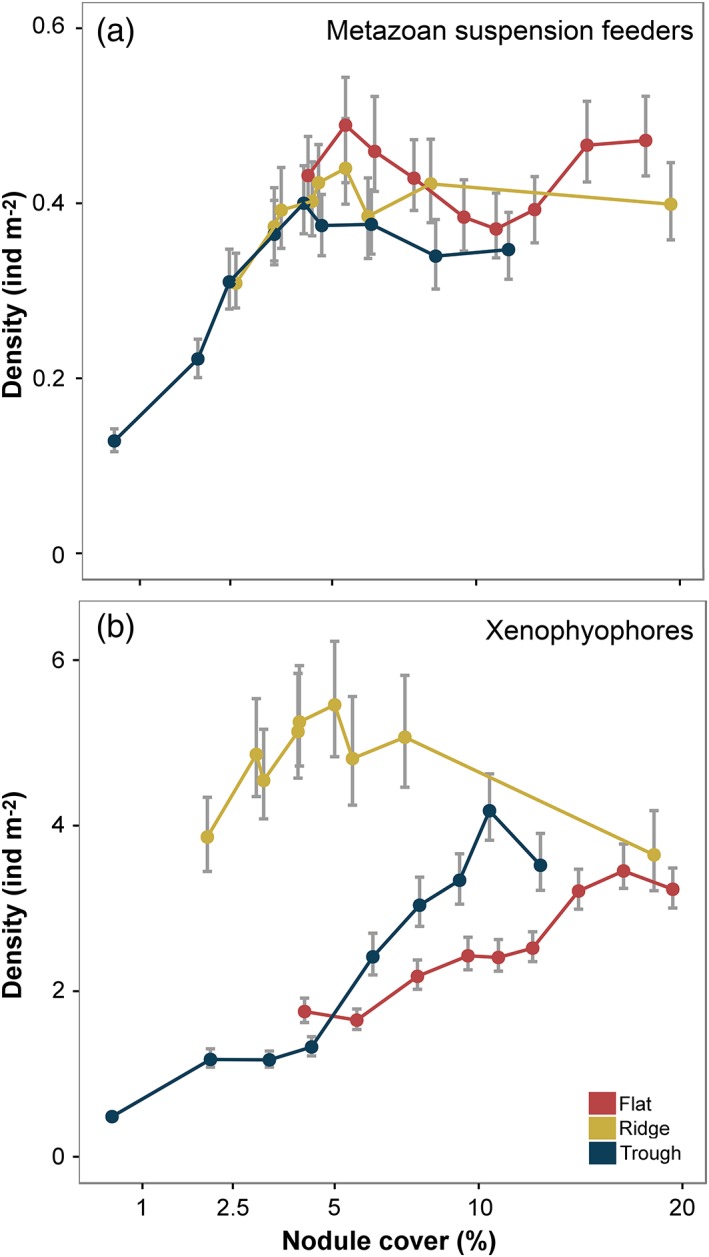
Examples of landscape‐type variation in faunal response to seafloor nodule cover. (**a**) Suspension‐feeding metazoans. (**b**) Xenophyophores. Data are mean numerical density values with 95% confidence intervals.

Xenophyophore density showed a rapid asymptotic relationship with nodule cover in the broad assessment but a different pattern in each landscape‐type, with a clearly higher abundance in the ridge (Fig. 6). Other studies have documented enhanced xenophyophore density on elevated terrain, e.g., seamounts (Levin and Thomas 1988; Wishner et al. 1990) and abyssal hills (Stefanoudis et al. 2016), and their dominance of the megafauna and high taxonomic diversity in the CCZ (Amon et al. 2016; Gooday et al. 2017). Although sediment‐dwelling species are well‐known, nodules clearly represent a very important habitat for xenophyophores (Gooday et al. 2015; Kamenskaya et al. 2015). While the specific feeding modes of xenophyophores remain uncertain (Gooday et al. 1993; Laureillard et al. 2004), the nodule‐attached forms are most likely suspension feeders, and the sediment‐dwellers most likely deposit feeders (Gooday et al. 2017). Our results suggest that, although nodule cover may limit the development of a part of the xenophyophore fauna (i.e., suspension feeders), landscape‐type variations are a stronger control on overall xenophyophore standing stock.

### Biological diversity

Perhaps, contrary to expectation (Amon et al. 2016, Vanreusel et al. 2016), there was little substantive variation in diversity along the nodule cover gradient studied (Fig. 2b; Table 1). Taxon richness (*S*_N_), taxon density (*S*_A_), and heterogeneity diversity, measured as the exponential Shannon index (exp *H*′), were not distinctly reduced in the minimal nodule cover class. Similarly, only taxon density exhibited a statistically significant correlation with nodule cover. In the context of nonimpacted natural assemblages sampled along a modest environmental gradient, these are not particularly unusual observations. However, they do suggest two cautions in connection with environmental impact assessment and monitoring in the deep‐sea polymetallic nodule‐mining context: (1) in conservation and preservation efforts, measured values of alpha diversity alone should not be relied upon in assessments of biological impact; and (2) the mensurative “experiments”, such as the present study, that only observe the natural state of the system can only provide “clues” as to potential impact. Consequently, it is important to assess additional parameters, not least standing stocks and faunal composition, when forming a view of potential environmental impact. Similarly, predictions made from mensurative studies ultimately require testing via controlled manipulative experiments (Caughley and Gunn 1996; Thiel 2001).

Heterogeneity diversity, measured as Simpson's index (1/*D*), was statistically significantly reduced in the minimal nodule cover class (Fig. 2b; Table 1). This may be consistent with the observation of Amon et al. (2016) that nodule cover does not need to be particularly high to promote higher megafauna diversity (although not necessarily richness) and with that of Vanreusel et al. (2016) in their suggestion that suspension feeder abundance drives the bulk of this change. The apparently reduced diversity in the minimal nodule‐cover class was attributable to the high numerical dominance of a single taxon (Porifera msp‐5). Note that, when assessed within‐landscape type, there was substantial disparity between heterogeneity diversity (both exp *H*′ and 1/D) measured in the ridge and trough environments in low nodule cover classes (~ 2.5%; Supporting Information Fig. S1‐2e–f). Structurally more complex habitats can be expected to provide a wider range of niches, promoting species coexistence and enhanced diversity in the deep‐sea benthos (Levin et al. 2001). Our results do suggest that nodules may act as “keystone structures” (Tews et al. 2004) in the regulation of habitat complexity at fine scales (tens of meters); however, landscape‐type variations do appear to modify the diversity response. In the present case, we might suggest that landscape (topographic) controls on bottom water flows and related patterns in benthic boundary layer particle concentrations and sediment deposition rates (Mewes et al. 2014; Peukert et al. 2018) may play an important role at these landscape scales (Simon‐Lledó et al. 2019).

### Faunal composition

Our data suggest that faunal composition changes continuously with nodule cover across the full spectrum of the gradient studied. The first step on that gradient (from nodule class 1 to 2) was, however, substantially greater than those that followed (Fig. 3). This initial “jump” is consistent with the change from an overwhelmingly background sedimentary habitat to a mosaic habitat with a varying admixture of nodule hard substrata to that sediment background. A higher dissimilarity of the lowest nodule‐class assemblage was somewhat expected, as most of the APEI6 megafaunal community (70% of taxa richness) were nodule‐dwelling taxa (Simon‐Lledó et al. 2019) with reduced abundance in the lowest nodule‐cover class (Supporting Information Fig. S2‐4). These taxa may simply not find enough suitable substratum to develop where nodules are limited, as typically occurs in the meiofaunal and macrofaunal assemblages (Mullineaux 1987; Veillette et al. 2007). The marked change in faunal composition from the first (~ 1%) to the second (~ 3%) nodule cover class indicates that even subtle increases in nodule availability can drive substantial variations in megafaunal communities (Amon et al. 2016). Nevertheless, variation in faunal composition appeared to be continuous from 3% to 20% nodule cover suggesting an ecological gradient (Fig. 3) and corresponding zonation of individual taxa (Fig. 4).

We found a clear shift in dominance from sponges (predominantly Porifera msp‐5) in the minimum nodule‐cover class to cnidarians at greater nodule cover, and within the latter, an alternation of dominance among primnoid soft corals, anemones, and bamboo corals with increasing nodule occurrence. This suggests that other environmental drivers may potentially covary along the nodule cover gradient. For instance, nodule size was positively correlated with nodule cover (*r*_s_ = 0.72, *p* < 0.001), with the surface area of nodules found in the lowest cover class (median: 1.66 cm^2^; IQR: 0.44) being almost half the size of those in areas with the highest coverage (median: 2.87 cm^2^; IQR: 0.42). Larger nodules are commonly found in areas with lower sediment accumulation rates and relatively stronger bottom‐current speeds (Skornyakova and Murdmaa 1992; Mewes et al. 2014). Variable development of particular deep‐sea suspension feeder populations can be regulated by bottom current speeds (Thistle et al. 1985; Smith and Demopoulos 2003) and also by the size of the available hard structures (Meyer et al. 2016), as has been previously determined for deep‐sea Alcyonacea (Watanabe et al. 2009). Areas with larger, and potentially more stable, nodules may provide a more suitable long‐term anchoring point for bamboo coral taxa, enabling a greater final colony height compared to, for example, primnoid soft corals (Lapointe and Watling 2015; Cairns 2016). In turn, the presumably stronger bottom current speeds in areas with large nodules perhaps limits the development of primnoids, which appear to find a suitable habitat in areas with comparably lower nodule availability (4–6%). Therefore, we hypothesize that factors inter‐related with nodule cover, like nodule size or bottom current speeds, possibly act as additional environmental filters, ultimately controlling population recruitment rates. We should of course note that little if anything is known of the natural history of these taxa in the CCZ, consequently the scope for biological interactions (e.g., interspecific competition) in determining these graded distributions is essentially unknown.

### Sustainable management and conservation

Our results suggest that areas less likely to be exploited by deep‐sea mining (i.e., low to intermediate nodule‐cover classes) would not necessarily serve the preservation of the full range of taxa that inhabit polymetallic nodule fields. Although these may act as source populations of taxa that do preferentially occur in high nodule abundance areas (i.e., Anthozoa or Bryozoa), our results suggest that they cannot support the abundant populations of these taxa found in high nodule cover areas (i.e., Isididae corals). Moreover, the potential deposition of sediment plumes in nondirectly exploited areas (Aleynik et al. 2017; Peukert et al. 2018) may also compromise the preservation of source populations for most suspension feeder taxa (Bluhm 2001). The latter represent the vast majority of the metazoan standing stock in the CCZ (Amon et al. 2016; Vanreusel et al. 2016) and appear to be the most sensitive to variations in nodule cover (i.e., this study). Consequently, the combined effects of nodule removal and sediment plume deposition may result in biodiversity and standing stock reductions at the landscape scale, with corresponding declines in the ecosystem services provided by megafauna.

Simplistically, a nodule field could be considered as two habitats: (1) the background sedimentary habitat and (2) the hard substratum environment of the nodules. At the physical scales inhabited by megafauna, the nodule field is likely better considered as a mosaic habitat comprising those two components. However, our results make clear that the mosaic habitat does not support a single biotope nor indeed two biotopes. Within the limits of the nodule cover gradient that we have been able to study, faunal composition exhibits continuous variation. Equally, it is clear that we do not yet fully understand the drivers of ecological variation along the nodule cover gradient. Consequently, sustainable management (Levin et al. 2016) and conservation plans (i.e., Smith et al. 2008), together with the monitoring programs that support them, must recognize this uncertainty if they are to be effective.

In closing, we should note that our primary analyses have concerned a broad assessment of nodule cover using data drawn from three distinct abyssal landscape types, all found within a single study area. These landscape‐scale variations in environmental and ecological characteristics (Simon‐Lledó et al. 2019; Supporting Information section S1) add an additional layer of complexity that can be expected to operate at the physical scale of individual conservation areas (APEI) and potential mining operation areas. However, it is not yet clear if the environmental conditions and faunas in the currently designated APEIs are similar to those of the mining claims and therefore it is not “safe” to assume their functionality. For instance, the range of nodule cover found in APEI6 (0–38%) possibly misrepresents those found in central CCZ locations, where nodules are typically larger and seafloor cover can reach higher levels (International Seabed Authority 2010). Further research, in other flats, ridges, and troughs and over wider nodule cover gradients in other CCZ areas, will still be needed to inform the design of preservation references zones within potential mining operation areas (Jones et al. 2018). It seems clear that these need to include appreciable areas of high nodule cover (e.g., prime mining target sites) and a range of landscape types if they are fully to achieve their conservation/preservation purpose.

## Conflict of Interest

None declared

## Supporting information

Appendix S1: Supplementary Information
