# Supplementary material for: Ecology of a polymetallic nodule occurrence gradient: Implications for deep‐sea mining
Source: Limnol Oceanogr. 2019 Mar 13;64(5):1883–94. doi: 10.1002/lno.11157 (PMC6774340; doi:10.1002/lno.11157)
Supplement: Supplementary file 1 — Appendix S1: Supplementary Information [file LNO-64-1883-s001.docx]

**Supporting Information**

# Section S1: Additional analyses within landscape type

The dataset was collected in three landscape types (LT), ‘Flat’, ‘Ridge’, and ‘Trough’ (main text, Fig. 1). To assess the potential influence of LT on ecological responses to the nodule cover gradient, we additionally carried out separate analyses within each LT. As in our overall analyses, images were ordered by nodule cover and divided into nine cover classes at breakpoints to yield an approximately equal number of megafauna specimens in each class. Megafauna data from each cover class, in each LT, was then subjected to a boostrap-like resampling procedure to produce 1000 samples targeting 250 specimens per sample. Faunal density and diversity measures (as in main text) were calculated for each boostrap-like subsample, and 95% confidence intervals derived by the simple percentile method (see main text).


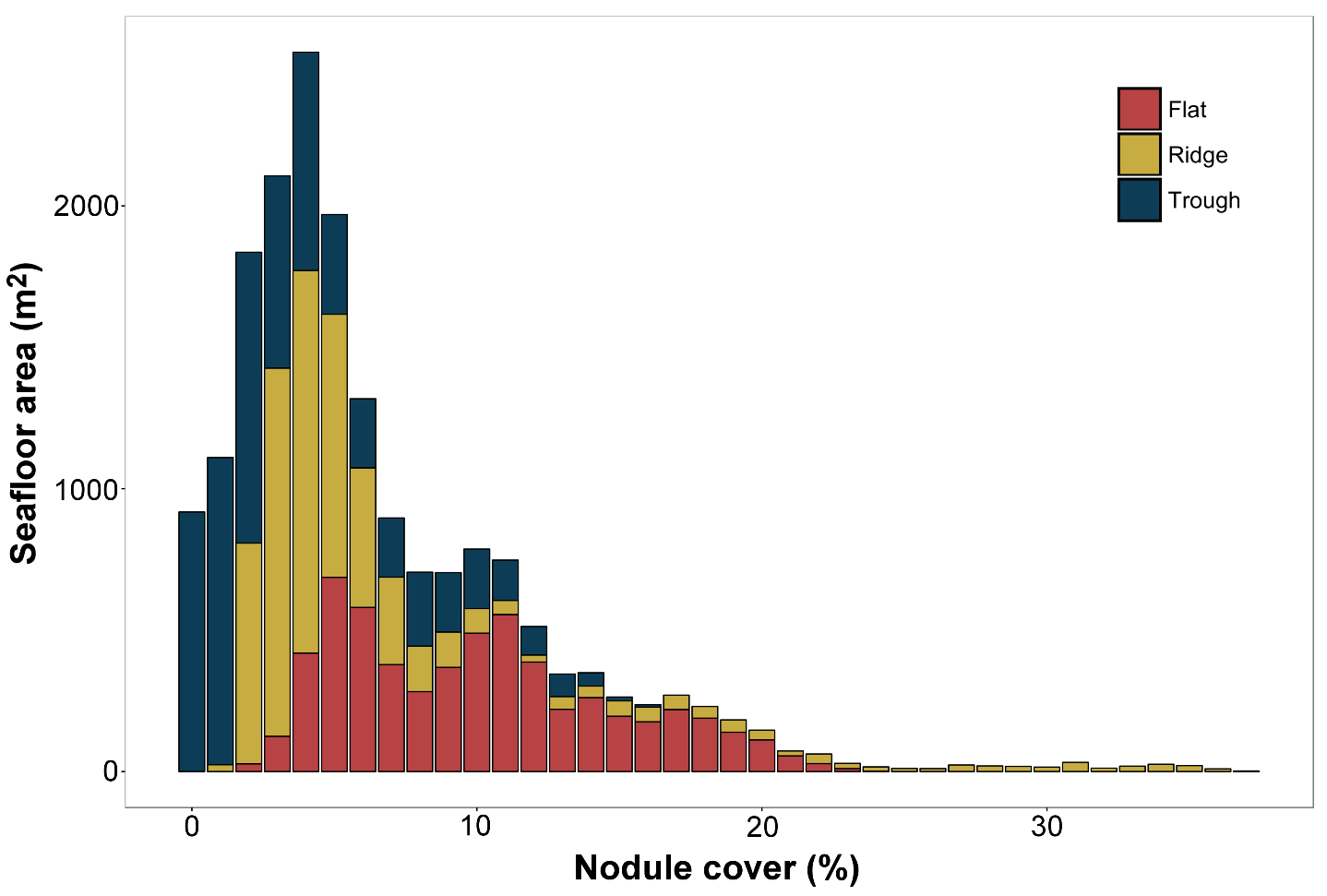


**Figure S1-1**. Areal distribution of nodule cover within each landscape type.


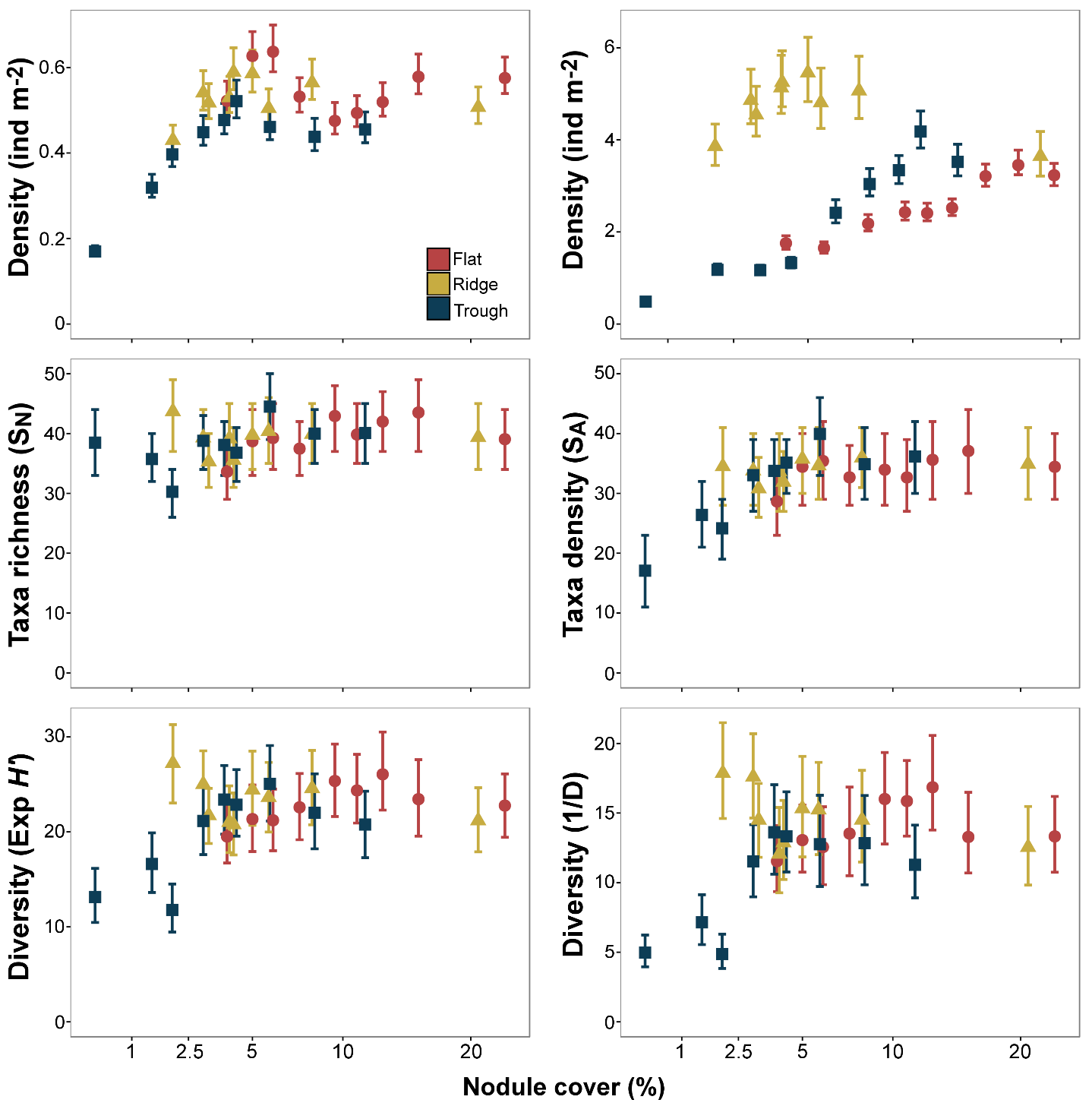


(f)

(e))

(d)

(c)

(b))

(a)

**Figure S1-2.** Variation of different ecological parameters across the nodule coverage gradient of each different APEI6 landscape type. Data are mean values of each parameter as calculated from each nodule cover class bootstrap-like sample set. Error bars represent 95% confidence. **(a)** Metazoan density. **(b)** Xenophyophore test density. **(c)** Morphospecies richness. **(d)** Morphospecies density. **(e)** Exponential Shannon index. **(f)** Inverse Simpson index.

# Section 2: Additional results of broad ecological assessment

#
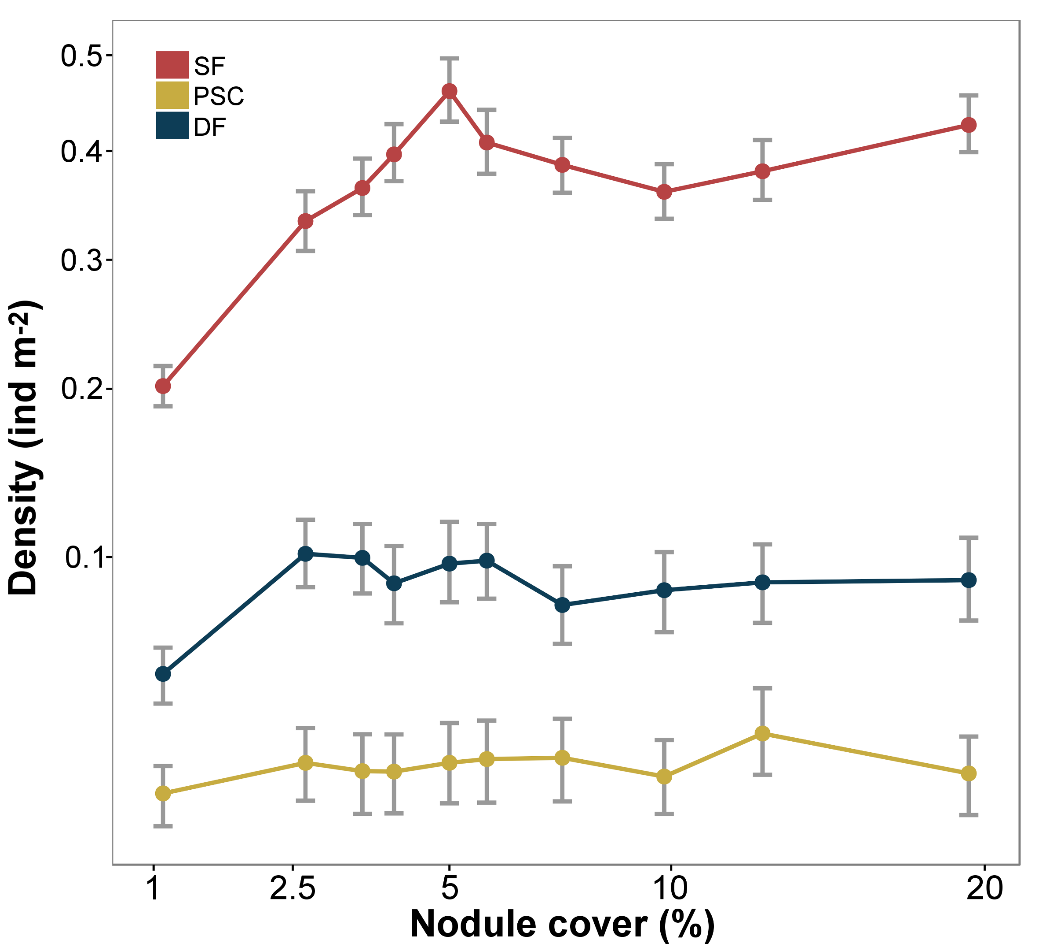


**Figure S2-1.** Variation in the density of three functional groups with nodule cover at the APEI6 seafloor. Data are mean density values of different metazoan types (SF: suspension feeders; PSC: predators and scavengers; DF: deposit feeders) calculated from each nodule cover class bootstrap-like sample set. Error bars represent 95% confidence intervals.


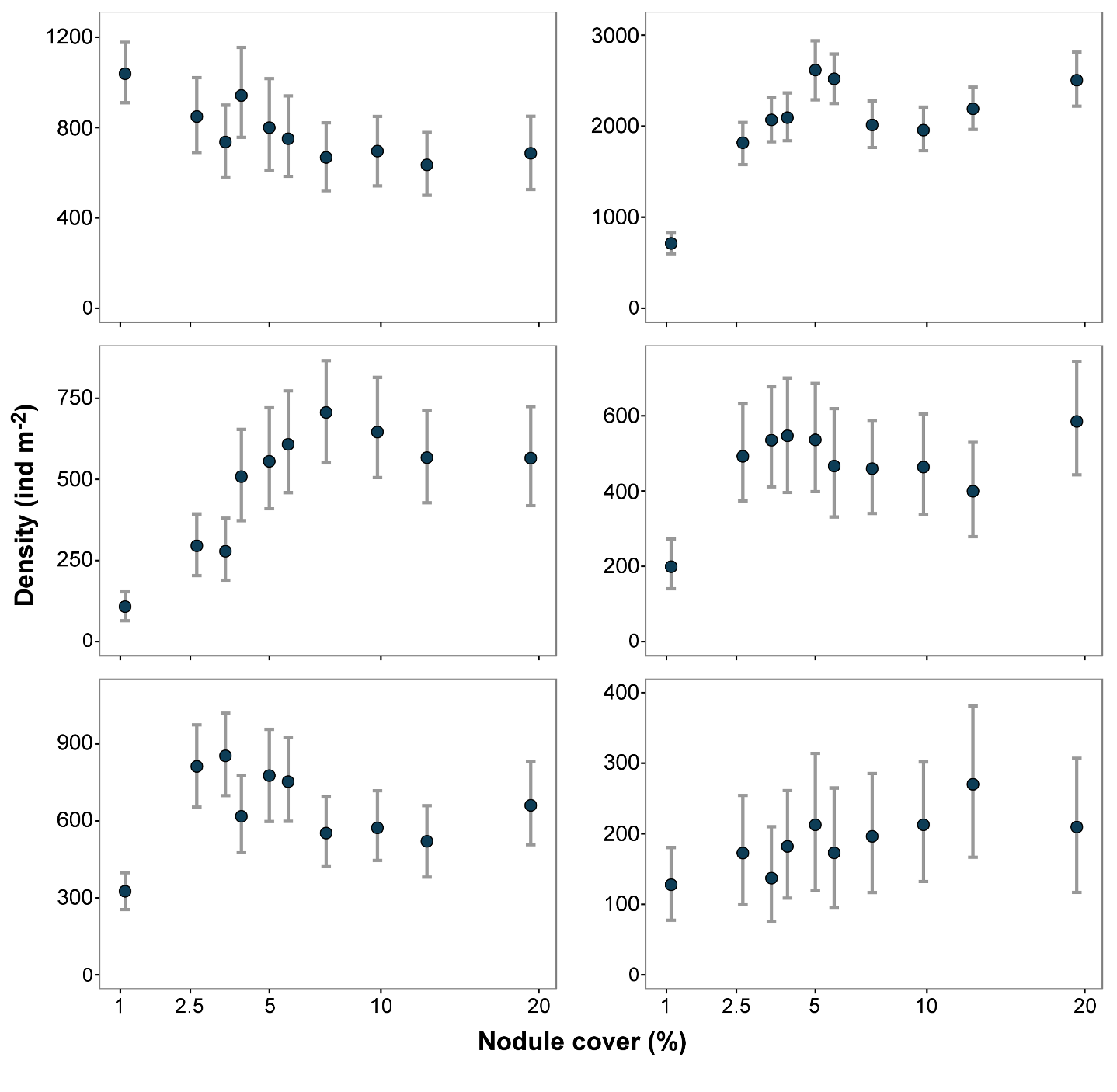


(c)

(e)

(f)

(b)

(d)

(a)

**Figure S2-2.**  Variation in the density of taxonomical groups with nodule cover. Data are mean density values of the six most dominant metazoan major taxa as calculated from each nodule-cover class bootstrap-like sample set. Error bars represent 95% confidence intervals. **(a)** Porifera. **(b)** Cnidaria. **(c)** Bryozoa. **(d)** Annelida. **(e)** Echinodermata. **(f)** Arthropoda.


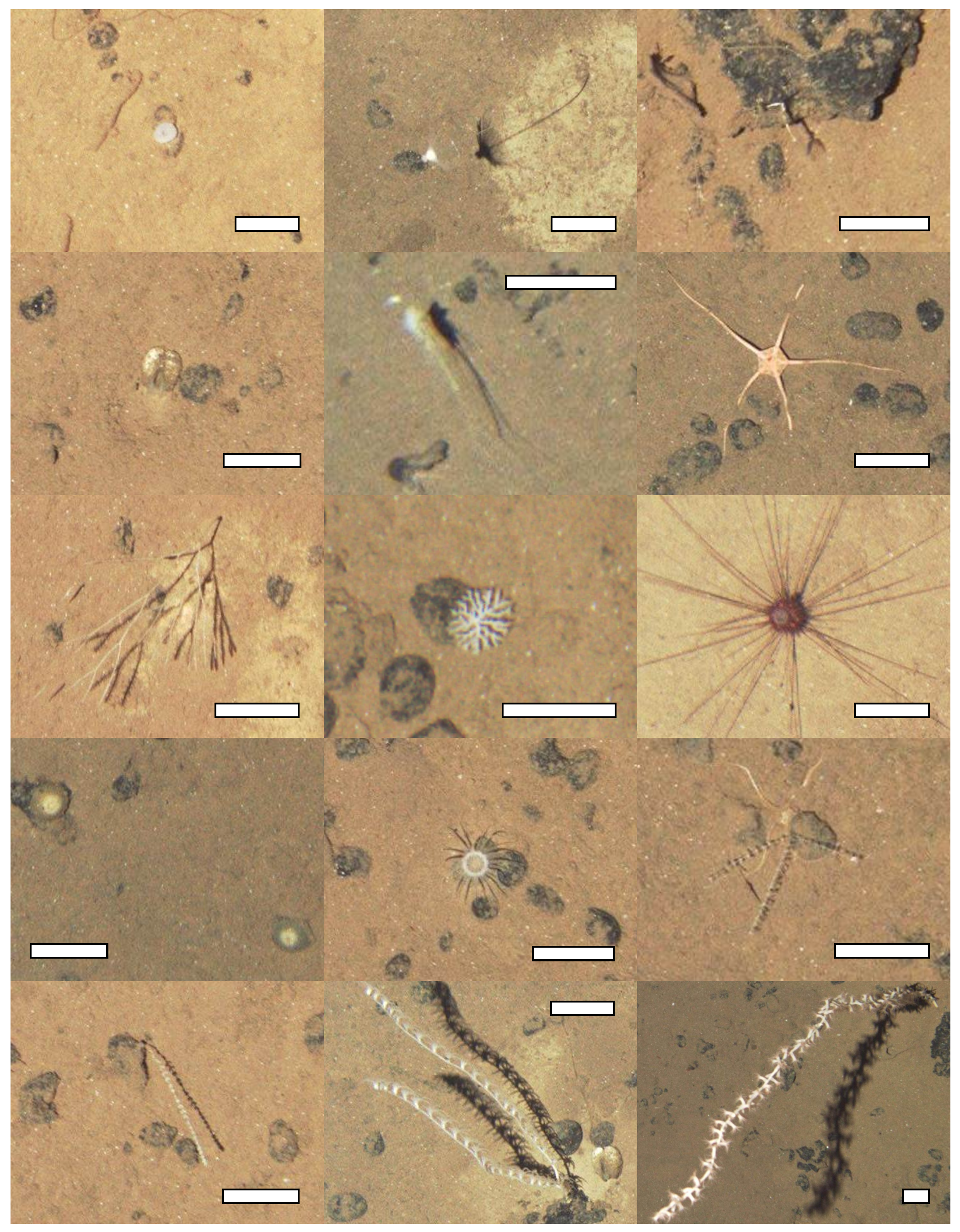


(o)

(n)

(m)

(l)

(k)

(j)

(i)

(h)

(g)

(f)

(e)

(d)

(c)

(b)

(a)

**Figure S2-3.** Top-15 most abundant metazoan morphospecies recorded at the APEI6 seafloor during JC120 AUV survey. Scale bars indicate 50 mm. **(a)** Porifera msp-5. **(b)** *Cladorhiza Mexicana* sp. inc. **(c)** Polychaete msp-5. **(d)** Irregularia msp-1. **(e)** Munnopsidae msp-2. **(f)** *Ophiosphalma* sp. indet. **(g)** *Columnella* sp. indet. (Bryozoa). **(h)** *Smithsonius* sp. indet. (Bryozoa). **(i)** Aspidodiadematidae genus indet. **(j)** Acitniaria msp-18. **(k)** Acitniaria msp-22. **(l)** *Callozostron* *bayeri* sp. inc. **(m)** *Calyptrophora* *persephone* sp. inc. **(n)** *Bathygorgia* *profunda* sp. inc. **(o)** *Lepidisis* sp. indet.


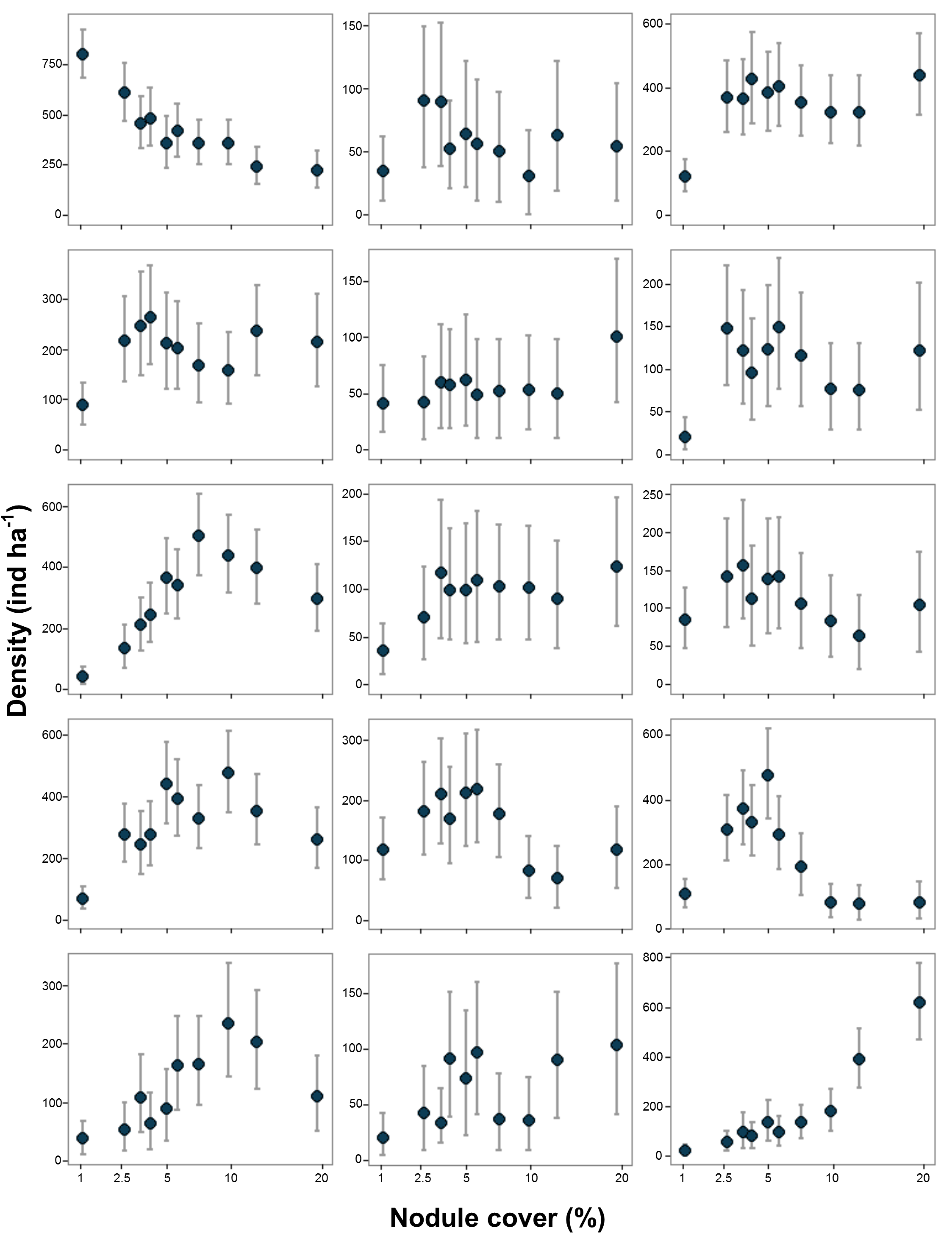


(o)

(n)

(m)

(l)

(k)

(j)

(i)

(h)

(g)

(f)

(e)

(a)

(d)

(c)

(b)

**Figure S2-4.** Variation in morphospecies density with nodule cover at the APEI6 seafloor. Data are mean density values of top-15 most abundant metazoan morphospecies as calculated from each nodule cover class bootstrap-like sample set. Error bars represent 95% confidence intervals. **(a)** Porifera msp-5. **(b)** *Cladorhiza Mexicana* sp. inc. **(c)** Polychaete msp-5. **(d)** Irregularia msp-1. **(e)** Munnopsidae msp-2. **(f)** *Ophiosphalma* sp. indet. **(g)** *Columnella* sp. indet. (Bryozoa). **(h)** *Smithsonius* sp. indet. (Bryozoa). **(i)** Aspidodiadematidae genus indet. **(j)** Acitniaria msp-18. **(k)** Acitniaria msp-22. **(l)** *Callozostron* *bayeri* sp. inc. **(m)** *Calyptrophora* *persephone* sp. inc. **(n)** *Bathygorgia* *profunda* sp. inc. **(o)** *Lepidisis* sp. indet.


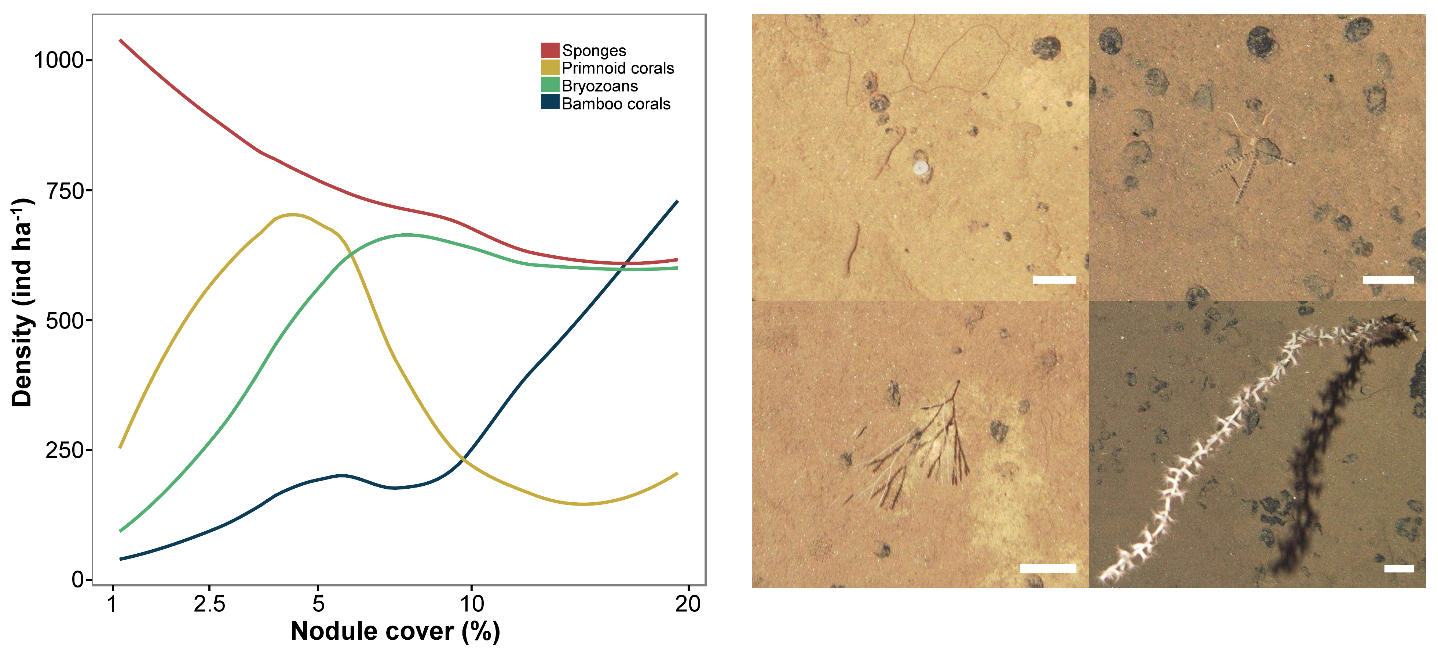


**Figure S2-5**. Variation in the density of selected metazoan taxonomic groups with nodule cover. Lines represent mean density values of each group as calculated from each nodule-cover class bootstrap-like sample set fitted by weighted least-squares, using a local polynomial regression.

**Table S2-1.** Spearman’s rank correlations of all ecological parameters with nodule cover. Summary results of tests performed between mean density (ind m^-2^) values of different metazoan groups as calculated from each nodule cover class bootstrap-like sample set and nodule cover variation, with detail of significant differences between nodule class 1 (mean cover = 1.1%) and the rest of classes (cover >2%). Distinct class 1: no overlap of class 1 confidence interval with any other class.

|  | **Distinct class 1** | **Correlations** | |
| --- | --- | --- | --- |
|  |  | r_s_ | p-value |
| ***Functional group*** |  |  |  |
| Deposit feeders | **yes** | -0.15 | 0.676 |
| Predators and scavengers | no | 0.28 | 0.425 |
| Suspension feeders | **yes** | 0.50 | 0.138 |
| ***Taxonomic Phylum*** |  |  |  |
| Annelida | **yes** | 0.10 | 0.777 |
| Bryozoa | **yes** | 0.78 | **0.008** |
| Cnidaria | **yes** | 0.49 | 0.150 |
| Arthropoda | no | 0.83 | **0.003*** |
| Echinodermata | **yes** | -0.25 | 0.489 |
| Porifera | no | -0.84 | **0.002** |
| ***Morphospecies*** |  |  |  |
| Polychaete msp-5 | **yes** | 0.18 | 0.627 |
| *Columnella* sp. indet | **yes** | 0.76 | **0.011*** |
| *Smithsonius* sp. indet | no | 0.50 | 0.138 |
| Acitniaria msp-18 | **yes** | 0.47 | 0.174 |
| Acitniaria msp-22 | no | 0.83 | **0.003** |
| *C. persephone* sp. inc | no | -0.36 | 0.310 |
| *B. profunda* sp. inc | no | 0.55 | 0.098 |
| *Lepidisis* sp. indet | no | 0.95 | **0** |
| *C.* *bayeri* sp. inc | no | -0.60 | 0.067 |
| Irregularia msp-1 | no | -0.03 | 0.934 |
| Aspidodiadematidae g. indet | no | -0.54 | 0.108 |
| *Ophiosphalma* sp. indet | no | -0.03 | 0.934 |
| Porifera msp-5 | no | -0.93 | **0** |
| *Cladorhiza.* *Mexicana* sp. inc | no | -0.24 | 0.511 |
| Munnopsidae msp-2 | no | 0.44 | 0.200 |
